# Genome-scale metabolic modeling and *in silico* analysis of opportunistic skin pathogen *Cutibacterium acnes*

**DOI:** 10.3389/fcimb.2023.1099314

**Published:** 2023-07-13

**Authors:** Su-Kyung Kim, Minouk Lee, Yi Qing Lee, Hyun Jun Lee, Mina Rho, Yunkwan Kim, Jung Yeon Seo, Sung Hun Youn, Seung Jin Hwang, Nae Gyu Kang, Choong-Hwan Lee, Seo-Young Park, Dong-Yup Lee

**Affiliations:** ^1^ School of Chemical Engineering, Sungkyunkwan University, Suwon, Gyeonggi-do, Republic of Korea; ^2^ Department of Biomedical Informatics, Hanyang University, Seoul, Republic of Korea; ^3^ Department of Computer Science, Hanyang University, Seoul, Republic of Korea; ^4^ R&D Center, LG Household & Healthcare (LG H&H), Seoul, Republic of Korea; ^5^ Department of Bioscience and Biotechnology, Konkuk University, Seoul, Republic of Korea

**Keywords:** skin microbiome, skin pathogen, *Cutibacterium acnes*, acne vulgaris, genome-scale metabolic model, Wood-Werkman cycle

## Abstract

*Cutibacterium acnes*, one of the most abundant skin microbes found in the sebaceous gland, is known to contribute to the development of acne vulgaris when its strains become imbalanced. The current limitations of acne treatment using antibiotics have caused an urgent need to develop a systematic strategy for selectively targeting *C. acnes*, which can be achieved by characterizing their cellular behaviors under various skin environments. To this end, we developed a genome-scale metabolic model (GEM) of virulent *C. acnes*, *i*CA843, based on the genome information of a relevant strain from ribotype 5 to comprehensively understand the pathogenic traits of *C. acnes* in the skin environment. We validated the model qualitatively by demonstrating its accuracy prediction of propionate and acetate production patterns, which were consistent with experimental observations. Additionally, we identified unique biosynthetic pathways for short-chain fatty acids in *C. acnes* compared to other GEMs of acne-inducing skin pathogens. By conducting constraint-based flux analysis under endogenous carbon sources in human skin, we discovered that the Wood-Werkman cycle is highly activated under acnes-associated skin condition for the regeneration of NAD, resulting in enhanced propionate production. Finally, we proposed potential anti-*C. acnes* targets by using the model-guided systematic framework based on gene essentiality analysis and protein sequence similarity search with abundant skin microbiome taxa.

## 1 Introduction

Acne vulgaris is a prevalent dermatological disorder that affects approximately 9.4% of the global population around 650 million adolescents and adults in the world (Chen et al., 2022). Although the exact cause of acne vulgaris is not fully understood, colonization by the opportunistic skin pathogen, *Cutibacterium acnes* (formerly known as *Propionibacterium acnes*) is a significant contributing factor (Dréno et al., 2020). *C. acnes* is a gram-positive and facultative anaerobic bacterium commonly found predominantly in human skin. Different ribotypes (RTs) of *C. acnes*, which can be classified based on their unique 16S rDNA sequences, have varying associations with healthy skin and acne vulgaris (Fitz-Gibbon et al., 2013). RT1, RT2, and RT3 are the most dominant RTs and can be found in both healthy and acne-affected skin. RT6 is predominant in healthy skin, while RT4, 5, 7, 8, 9, and 10 are associated with acne vulgaris. Overgrowth and imbalances of types of *C. acnes* can cause inflammation together with a loss of skin microbiome diversity, leading to the development of pimples (Dréno et al., 2018; Dréno et al., 2020). In this regard, a clinical trial (NCT03709654) was previously conducted on a live biotherapeutic for the treatment of acne vulgaris in order to eliminate disease-associated *C. acnes* strains *via* a delivery of health-associated strains to restore the skin into a healthy state (Vargason and Anselmo, 2021).

Another characteristic of *C. acnes* strains includes the production of pro-inflammatory short-chain fatty acids (SCFAs) as glycolytic end products, especially propionate. Although SCFAs have beneficial effects on skin health (Christensen and Brüggemann, 2014; Nakamura et al., 2020), they may also stimulate free fatty acid receptors, thereby triggering inflammatory reactions in skin immune cells (Sanford et al., 2019). Particularly, propionate, one of the major SCFAs, may increase cytotoxic effects by inducing pH changes (Tax et al., 2016), and provoke the immune response *via* interaction with the Toll-like receptors (TLR), e.g., TLR2 and TLR4 (Kim et al., 2002; Nagy et al., 2005). Similarly, *C. acnes* produces two types of porphyrins which give rise to the secretion of proinflammatory cytokines by activating the NLRP3 (NOD-, LRR- and pyrin domain-containing protein 3) inflammasome (Sanford et al., 2019; Josse et al., 2020; Spittaels et al., 2021). In addition, *C. acnes* proliferation can be promoted by secreted triacylglycerol (TAG) lipase which degrades sebum lipids into free fatty acids and metabolizable glycerol for obstructing pilosebaceous unit and subsequently inducing its anaerobic growth (Sanford et al., 2019; Josse et al., 2020; Spittaels et al., 2021). Interestingly, acne-associated strains produce significantly higher amounts of the aforementioned biomolecules, i.e., propionate, porphyrins, and TAG lipase, that contribute to the development of acne vulgaris, compared to other commensal strains (Higaki et al., 2000; Johnson et al., 2016; Borrel et al., 2019). Thus, such opportunistic behavior of virulent *C. acnes* can be explained by the collective effects of these biomolecules that are conditionally synthesized and produced under different skin and culture environments. For example, the HL045PA1 strain belonging to phylotype IA-1 expressed TAG lipase and uroporphyrinogen III synthase, an essential enzyme in porphyrin metabolism, only in a sebum-like medium that mimics the sebaceous gland environment (Borrel et al., 2019).

Typically, the first choice for treating acne vulgaris is the empirical use of antibiotics, but it could induce antibiotic-resistance and skin disease-associated dysbiosis (Goodarzi et al., 2020; Karadag et al., 2021; Pessemier et al., 2021). Thus, there is an urgent need to develop a more rational strategy for selectively targeting relevant skin pathogens, which could be achieved by characterizing the cellular behaviors under various skin environments and identifying their virulence factors. In this regard, one of promising approaches is flux balance analysis (FBA) with a strain-specific genome-scale metabolic model (GEM), allowing us to capture the condition-dependent metabolic states based on their gene-protein relationship (Orth et al., 2010). This approach has been successfully applied to other common human pathogens, including *Pseudomonas aeruginosa*, *Klebsiella pneumoniae*, and *Acinetobacter baumannii*,to portray their unique virulent behavior and suggest possible antimicrobial agents (Bosi et al., 2016; Henry et al., 2017). For example, *P. aeruginosa* GEM prediction in synthetic cystic fibrosis medium elucidated the metabolic connectivity of virulence factor synthesis with central metabolism, thereby suggesting homoserine dehydrogenase as a potential target for selectively reducing virulence factor synthesis with an experimental validation (Bartell et al., 2017). In addition, metabolic states of highly virulent strain (KPPR1) of *K. pneumoniae* were compared with low-virulence stain (MGH 78578) under rich nutrient conditions using the GEMs, identifying antimicrobial targets such as thymidylate kinase and lipid A disaccharide synthase (Henry et al., 2017). For *A. baumannii*, its GEM was combined with transcriptomic data to describe the flux changes in central metabolism after antibiotic treatment (Presta et al., 2017). In a previous study (McCubbin et al., 2020), metabolic networks of five *Propionibacterium* species, including *P. acnes*, were constructed through a pan-genomic analysis of 16 *Propionibacterium* genomes. Building upon this prior work, our study aims to further enhance the understanding of the cellular metabolism and virulence of *C. acnes* within the skin environment from pathogenic point of view. To do so, we reconstructed a comprehensive GEM of acne-associated *C. acnes* and performed *in silico* growth simulations, as such revealing the underlying mechanism of cell growth and pathogenicity under endogenous carbon sources, and lastly suggesting potential targets in acne vulgaris treatments that selectively reduce their population.

## 2 Materials and methods

### 2.1 Genome-scale metabolic network reconstruction

The genome-scale metabolic model (GEM) of *C. acnes*, *i*CA843, was reconstructed based on the established procedures (Thiele and Palsson, 2010). To reconstruct the GEM of a virulent *C. acnes* strain, the genome sequences and corresponding annotation of HL043PA1 from National Center for Biotechnology Information (NCBI) database as of 22 March 2022. HL043PA1 is a strain belonging to ribotype 5, which is known to be associated with acne vulgaris. The preliminary metabolic network draft was built using CarveMe (Machado et al., 2018). The nomenclature of metabolites and reactions was based on the BiGG database (Schellenberger et al., 2010). Next, additional metabolic and transport reactions of *C. acnes* were annotated by EggNOG 5.0 (Huerta-Cepas et al., 2019) and BlastKoala (Kanehisa et al., 2016), with various database such as BiGG (King et al., 2016), KEGG (Kanehisa, 2000), SEED (Henry et al., 2010), UniProt (Bateman et al., 2021), and TransportDB (Elbourne et al., 2017). Then, the relevant metabolic reactions were included with the corresponding gene-protein-reaction (GPR) assignments based on either direct or indirect biochemical evidence from the biochemical databases and literature. An effort was made to annotate metabolic gap-filled reactions that lacked GPR annotation, and in cases where GPR information was unavailable, the gap-filled reactions were excluded from the model. To enhance the reliability of each reaction in the model, a comparison of protein sequences using BLASTp (Altschul et al., 1990) was conducted prior to their inclusion with e-values< 1 × 10^-50^ and a percentage identity >70%. The reversibility of coupled reactions was corrected based on the biochemical information from literature and online databases such as MetaCyc (Caspi et al., 2014), Brenda (Chang et al., 2021), Virtual Metabolic Human (VMH) (Noronha et al., 2019), and eQuilibrator (Beber et al., 2022), to comprehensively consider the physiological direction and biochemical thermodynamics of the reactions. Next, the model leveraged with previously reconstructed metabolic model of *P. acnes* 6609 (McCubbin et al., 2020) to cross-check the reactions with GPR associations and further expand the energy metabolism. To do this, homologous proteins between the HL043PA1 and 6609 strains were identified based on BLASTp, and the metabolic reactions associated with the resulting homologous proteins were inspected manually based on literature and gene annotation. Only reactions confirmed to have protein homology and literature evidence, and a valid GPR were added to the current reconstruction. For common reactions between the current and previous reconstruction, the GPR of these reactions was compared and updated accordingly if necessary. In particular, for differing reactions between the models, extensive research was conducted based on protein homologs, biochemical databases, and literature to determine whether to include these reactions, and they were included in the current model only if biological evidence was present. At the very last step, the quality of reconstructed GEM was evaluated by comparing it with relevant experimental data and a metabolic model test suite called MEMOTE (Lieven et al., 2020), which is a community standard for this purpose.

### 2.2 Biomass equation formulation

We derived a biomass equation for *C. acnes* with information of macromolecular and monomeric composition information obtained from published data (see **Supplementary Data Sheet 1**). Note that we also partially used biomass composition data from a taxonomically close species in the case where *C. acnes* specific data is unavailable (Rocha et al., 2008). Note that the macromolecular composition and some parts of the monomeric composition including lipid, polysaccharide and small molecules, were referred from the *Propionibacterium* biomass equation derived from the previous model (McCubbin et al., 2020), since taxonomically related species are shared among *Propionibacterium*. The protein, DNA and RNA compositions of *C. acnes* were estimated based on the genome sequence data used in the model reconstruction, while the fatty acid composition of *C. acnes* was obtained from literature (Moss et al., 1967). The growth and non-growth associated maintenance (GAM) were calculated based on the macromolecular composition, and non-growth associated maintenance (NGAM) were assumed to be identical to that of *P*. *acidipropionici* (Zhang et al., 2015).

### 2.3 Constraint-based flux analysis

The metabolic behavior of *C. acnes* was simulated under different conditions using constraint-based FBA (Raman and Chandra, 2009; Orth et al., 2010). All constraint-based flux analysis simulations were carried out using COBRA Toolbox in MATLAB R2020a (Schellenberger et al., 2011) with CPLEX optimization solver. The constraints used in model simulations are provided in **Supplementary Table 1**. The cell was set to maximize biomass objective function ( $v_{biomass}$ ) while constraining uptake rates of other nutrients, such as uptake rates of carbon source and other complex medium components. The glucose and glycerol uptake rate were constrained at 10 mmol gDW^-1^ h^-1^ and 20 mmol gDW^-1^ h^-1^, respectively. Mathematical representation of the optimization problem can be expressed as follows:


$$max\;v_{biomass}$$

$$\sum_{j}S_{ij}v_{j}=\;0$$

$$v_{j}^{min}\leq v_{j}\leq \;v_{j}^{max}$$

Where $S_{ij}\;$ is the stoichiometric coefficient of metabolite *i* that participates in reaction *j* and $v_{j}$ is the flux of reaction *j*. Reaction constraints were given by assigning lower and upper bounds to the reaction ( $v_{j}$ ) as $v_{j}^{min}$ and $v_{j}^{max}$ , respectively.

We predicted vitamin auxotrophy by constraining the uptake rate of each vitamin to zero and maximizing the biomass objective function. Vitamin auxotrophy was defined as over a 90% decrease in growth when the corresponding vitamin was excluded from the media (Koduru et al., 2017). SCFA production rate and intracellular flux distributions in glucose and glycerol were simulated using a variation of parsimonious FBA (pFBA) that optimizes the objective function and sequentially minimizes the flux through the model (Lewis et al., 2010). The turnover rates of metabolites under the aforementioned conditions were described using flux-sum, as in previous studies (Chung and Lee, 2009; Mishra et al., 2016). Assuming that the cell is in steady-state flux balanced condition, the flux-sum for metabolite i , $\phi_{i}$ , is given by:


$$\phi_{i}=\frac{1}{2}\sum_{j}^{\;}\left|S_{ij}v_{j}\right|$$

The sum of all fluxes containing metabolite i will give the total rate of consumption and generation, having this value will give the turnover rate of metabolite i . The flux and flux-sum intensity were also calculated in our study. Flux intensity was obtained by normalizing the flux values of each reaction by the maximum flux value of the reaction across the conditions, while flux-sum intensity for each reaction was obtained by dividing them by the corresponding maximum flux-sum value of the metabolite across the conditions. The flux and flux-sum values for both glucose and glycerol conditions are provided in **Supplementary Table 2**.

### 2.4 Identification of specific antimicrobial targets in *C. acnes*

As potential antimicrobial targets for *C. acnes*, essential genes of *C. acnes* in glycerol condition were identified by using the single gene deletion function provided in the COBRA toolbox (Joyce and Palsson, 2007). Genes whose knockouts resulted in a predicted growth rate of less than 10% of the wild-type predicted growth rate were considered essential. To sort out the essential genes in *C. acnes* that are homologous to other skin microbe gene sequences, the essential genes were further subjected to a BLASTp protein sequence similarity search against 180 abundant skin bacteria taxa present at > 0.1% of the reads in at least one sample among 251 collected samples (Bewick et al., 2019). The list of the abundant microbiome taxa is provided in **Supplementary Table 3**. The whole genome sequences of reference strains of abundant skin microbiome taxa were retrieved from NCBI and used for BLASTp analysis. The protein sequences with an e-value < 1 × 10^-50^ were considered homologs (Heinken et al., 2023).

### 2.5 Reconstruction of ribotype-specific GEMs for RT1 and RT6

Distinct associations have been observed between different ribotypes (RTs) of *C. acnes* and acne vulgaris (Fitz-Gibbon et al., 2013), indicating that exploring the metabolic differences among RTs may provide new insights into acne vulgaris. To investigate the variations in metabolic and phenotypic behavior among RTs, we focused on two specific RTs for GEM reconstruction and analysis: RT1, which is highly prevalent in both affected and healthy skin, and RT6, which is associated with healthy skin. The genome sequences of *C. acnes* strains ATCC6919 and HL110PA3 were utilized for the reconstruction of RT1 and RT6 GEMs, respectively. These genome sequences were retrieved from the NCBI database as of 23 February 2023.

The RT-specific GEMs were built based on protein homology and the reconstructed *i*CA843 model. SonicParanoid (Cosentino and Iwasaki, 2019) was utilized in its default mode to search for homologous proteins between ATCC6919 (RT1) and HL043PA1 (RT5), as well as between HL110PA3 (RT6) and HL043PA1 (RT5). The identified homologous proteins were used to transfer reactions with appropriate GPR associations from *i*CA843 to the RT1 or RT6 GEMs. Reactions lacking GPR information were automatically transferred to other GEMs if they were necessary for the growth or metabolic phenotypes of *C. acnes*. Reactions associated with RT-specific proteins were identified based on the draft GEMs reconstructed using ModelSEED (Seaver et al., 2021) and CarveMe (Machado et al., 2018) with manual curation.

### 2.6 Comparative genomics of *C. acnes* ribotypes

To identify orthologous and ribotype-specific genes within the *C. acnes* strains, a total of 167,830 protein sequences was compiled from 73 strains representing different ribotypes. These sequences were then subjected to clustering using CD-HIT program (version 4.8.1) with an amino acid similarity threshold of 70% (Fu et al., 2012). From the pool of dispensable genes found in two or more strains, we specifically retained the genes that were unique to RT1, 5, or 6.

## 3 Results

### 3.1 Genome-scale metabolic model reconstruction of propionate-producing *C. acnes*

**Figure 1:**
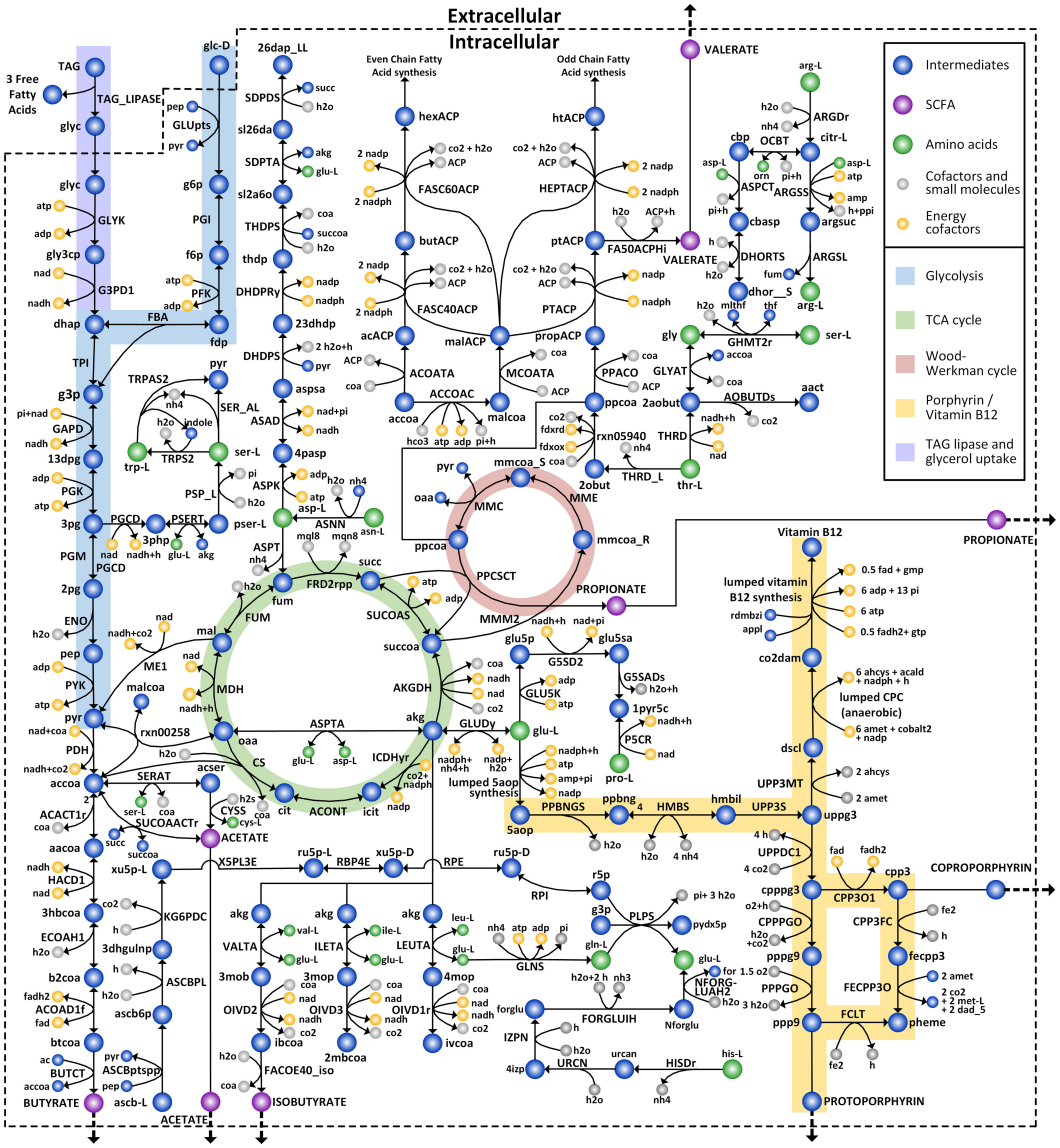
*C. acnes* metabolic network of *i*CA843. *C. acnes* GEM includes central carbon metabolic pathways and amino acid biosynthesis pathways, in addition to characteristic pathways, such as the Wood-Werkman cycle, the SCFAs fermentation pathways, the porphyrin and vitamin B12 biosynthesis pathways, as well as skin condition related TAG lipase reaction and glycerol uptake pathway.

The GEM for *C. acnes* HL043PA1, *i*CA843, was reconstructed following procedures (see Materials and Methods). Initially, we build a draft metabolic network based on the functional annotation of genes from the whole genome sequence of the HL043PA1 strain. This strain belongs to RT5, which is reported to be strongly associated with acne vulgaris (Fitz-Gibbon et al., 2013; Barnard et al., 2016). The network was then manually checked to identify any discrepancies between the network and known physiological metabolism. In this step, it is important to rectify incorrect GPR relations by considering species-specific enzyme annotation and include the relevant metabolic reactions which may not be functionally assigned due to the limitation of homology-based approaches based on biochemical databases and literatures. As an example, we updated the information regarding methylmalonyl-CoA carboxyltransferases (MMC; EC 2.1.3.1), a critical reaction in the propionate biosynthesis. We made the necessary modification to the GPR annotation of MMC by newly incorporating a previously unannotated subunit that was identified as a hypothetical protein lacking specific functionality in the NCBI annotation. We also added type-2 phosphatidic acid phosphatase (EC 3.1.3.4), which was not included initially in the draft model, according to the literature reporting that it is required for *de novo* synthesis of phosphatidylglycerol in phospholipid metabolism (Kanoh et al., 1999). As a result, the GPR of 198 reactions were updated and 392 metabolic reactions were newly included in the model, based on the functional annotation, biochemical databases, and experimental evidence. In addition, a total of 705 metabolic reactions were excluded from the draft model due to redundancy in fatty acid and phospholipid metabolism, as well as the presence of periplasmic reactions that are not feasible in Gram-positive bacteria such as *C. acnes*. Next, the reconstructed model was further expanded by integrating existing metabolic model of *P*. *acnes 6609* (McCubbin et al., 2020), which was built based on the pan-genome of *Propionibacterium* and offered novel metabolic insights into the energy conservation mechanism. This process led to an update of GPR for 297 existing reactions, deletion of 94 misannotated reactions, and incorporation of 163 new reactions, including a novel ferredoxin-based energy conservation mechanism of *Propionibacterium* proposed in previous study. Reversibility of all reactions were cross-checked based on several databases such as MetaCyc (Caspi et al., 2014), Virtual Metabolic Human (VMH) (Noronha et al., 2019), and eQuilibrator (Beber et al., 2022), which prevents biologically unfavorable intracellular fluxes in model simulation. In addition, we formulated a biomass equation which was derived from the macromolecular and monomer compositions as described in Material and Methods, and **Supplementary Data Sheet 1**. Finally, the resulting model, *i*CA843, comprises 843 genes, 1510 reactions and 1194 metabolites, covering comprehensive central carbon, amino acid, and lipid metabolisms, as well as relevant biosynthetic pathways of key virulence factors such as SCFAs, TAG lipase and various types of porphyrins (**Figure 1**). The list of reactions, along with their respective gene associations and metabolites, is provided in both systems biology markup language (SBML) and excel format (**Supplementary Data Sheet 2**). Additionally, we confirmed the network consistency of *i*CA843 using the online tool MEMOTE (Lieven et al., 2020), achieving an overall score of 89% (**Supplementary Data Sheet 3**).

**Figure 2:**
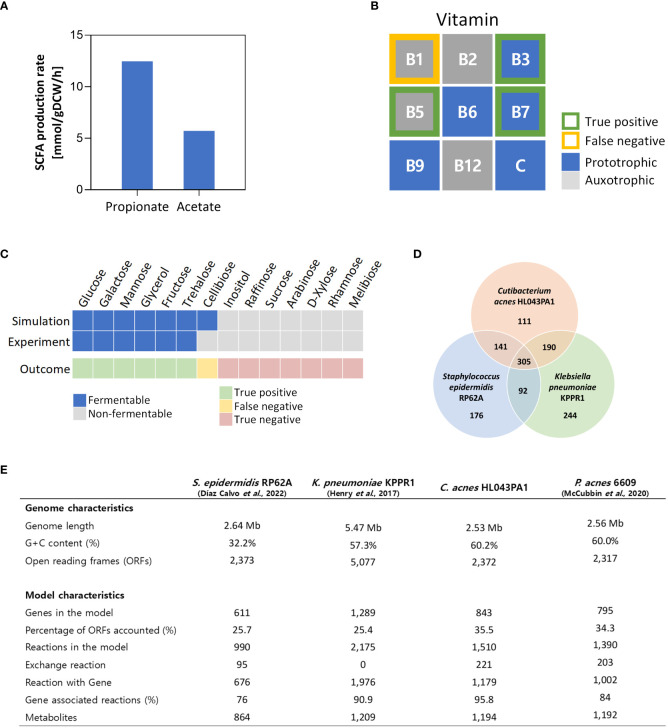
Qualitative model validation and comparison with other GEMs acne-inducing skin pathogens. **(A)** Predicted fermentative production pattern of propionate and acetate in rich media with glucose using *i*CA843. **(B)** Auxotroph simulations for vitamins B and C **(C)** Fermentable substrate phenotyping for various carbon sources and comparison with reported experimental data by Puhvel (1968). **(D)** Venn diagram compares the EC numbers of the GEMs of the acne-inducing skin bacteria, including *C. acnes* HL043PA1, *Staphylococcus epidermidis* RP62A, and *Klebsiella pneumoniae* KPPR1. **(E)** The genome and metabolic network characteristics of the skin bacterial species.

Next, we assessed the quality of *i*CA843 by simulating the cell growth under glucose condition. Remarkably, the predicted fermentative behavior was highly consistent with the reported SCFA measurements (Stowers et al., 2014), with propionate producing more than twice that of acetate (**Figure 2A**). It should be noted that the secretion of propionate and acetate was not constrained in the FBA simulation (see Materials and Methods), showing that *i*CA843 successfully captures the physiological and metabolic traits of *C. acnes*, a representative propionic acid bacteria (PAB) that efficiently ferments carbon sources to produce propionate through the Wood-Werkman cycle. The Wood-Werkman cycle consists of MMC, propionyl-CoA:succinate CoA transferase, and methylmalony-CoA mutase (Bücher et al., 2021). Next, we performed simulations to determine the essential vitamins B and C required for cell growth, which identified 4 auxotrophic vitamins (thiamin, riboflavin, pantothenate, and cobalamin) and 5 prototrophic vitamins (nicotinamide, pyridoxine, biotin, folate, and ascorbate) (**Figure 2B**). Notably, our results are consistent with experimental observations for 3 vitamins, namely nicotinamide, pantothenate, and biotin (McDowell et al., 2016), while there is a discrepancy between our simulation results (thiamin as essential for growth) and the experimental evidence. This inconsistency suggests the necessity for further investigation to reconcile the differences and gain a deeper understanding of the role of thiamin in the growth of *C. acnes*. Upon conducting a protein sequence similarity search targeting known enzyme sequences for thiamin biosynthesis, a complete biosynthesis pathway for thiamin could not be identified, making it impossible to gap-fill the related pathway. This finding suggests the presence of potential knowledge gaps in the genome annotation or an alternative, undiscovered biosynthesis pathway, which may explain the HL043PA1-specific auxotrophic behavior. In addition, we conducted fermentable substrate phenotyping for various carbon sources, and compared *in silico* predictions with experimental data reported by Puhvel (1968), giving rise to the growth phenotypes which are in good agreement with 13 out of 14 different carbon sources available naturally under human skin environment or provided from skin care products (**Figure 2C**).

To provide an overview of *C. acnes* metabolic traits as a skin inhabitant and to highlight the uniqueness of *C. acnes* metabolism in relation to acne vulgaris, we compared *i*CA843 with GEMs of other skin bacteria that have been reported to contribute to acne vulgaris pathogenesis, *Staphylococcus epidermidis* and *Klebsiella pneumoniae* (**Figure 2D**) (Kumar et al., 2016; Henry et al., 2017; Díaz Calvo et al., 2022). All three species can utilize glycerol, a carbon source abundantly available in the skin environment that provokes bacterial fermentation (Balasubramaniam et al., 2020). They also commonly produce protoporphyrin IX, which is a precursor to heme, as well as several SCFAs such as acetate and butyrate. However, in contrast, *C. acnes* can uniquely synthesize coproporphyrin III, which is more relevant to acne lesions than protoporphyrin IX (Patwardhan et al., 2017), and utilize distinctive SCFA biosynthetic pathways to produce propionate and acetate as major metabolic byproducts through the Wood-Werkman cycle and the newly recognized succinyl-CoA:acetate CoA-transferase (SUCOAACTr) (Zhang et al., 2021), respectively. On the other hand, *K. pneumoniae* produce propionate from the propanediol pathway (Luo et al., 2012), while *S. epidermidis* has no metabolism for its synthesis. Furthermore, an analysis of the genome and model characteristics of the three skin pathogens revealed that although the *C. acnes* model has the shortest genome length and the fewest open reading frames, it also has the highest percentage of accounted ORFs in the model and gene-associated reactions (**Figure 2E**). In addition, a comparison between the genome and model characteristics of *i*CA843 and the previous *P. acnes* 6609 GEM showed that *i*CA843 encompasses a greater proportion of open reading frames and gene-associated reactions. The inclusion of a higher percentage of gene-supported reactions and a more comprehensive set of network components in *i*CA843 has significantly expanded the metabolic capabilities of the model. This expansion is expected to capture specific metabolic characteristics unique to *C. acnes*.

### 3.2 Characterization of physiological behaviors and metabolic states of *C. acnes* under endogenous carbon sources in human skin

**Figure 3:**
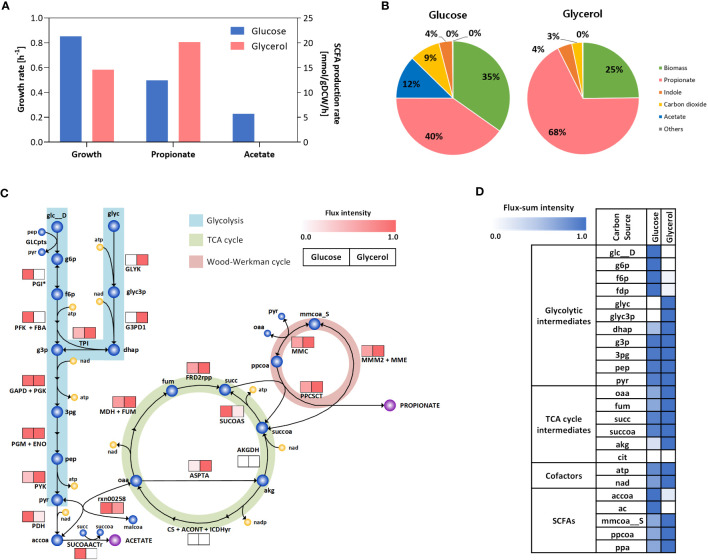
*In silico* phenotype predictions under endogenous carbon sources in human skin. **(A)** Predicted rate of growth and SCFAs production. **(B)** The predicted breakdown of carbon output from each carbon source. **(C)** Flux map showing the intracellular flux distribution across glycolysis, TCA, Wood-Werkman cycle and SCFA biosynthesis pathways. **(D)** Heatmap representing the flux-sum intensity of SCFAs, cofactors and others in central metabolism. In **(C, D)**, the color intensity corresponds to the flux or flux-sum values normalized with respect to the maximum value observed for each reaction or metabolite, respectively. The abbreviations of enzymes and metabolites is provided in **Supplementary Table 4**.

*C. acnes* exhibits a significant fermentative characteristic by producing a substantial quantity of propionate through the Wood-Werkman cycle. This ATP-independent pathway efficiently converts pyruvate into oxaloacetate (Wang et al., 2015). In addition to propionate, *C. acnes* also produces other SCFAs, including acetate. These SCFAs play a role in stimulating free fatty acid receptors and can trigger inflammatory reactions in skin immune cells (Sanford et al., 2019), while propionate can have cytotoxic effects by causing pH changes (Tax et al., 2016), and can also provoke an immune response (Kim et al., 2002; Nagy et al., 2005). The production of SCFAs by *C. acnes* contributes to the complex interplay between the microbiota and the host immune system in the context of skin health and inflammation. Therefore, our aim is to elucidate the metabolic flux distributions attaining to these fermentative behaviors in human skin, as an effort to gain a better understanding of the underlying intercellular mechanisms involved in the development of acne vulgaris. We performed flux simulations under anaerobic conditions to mimic the environment of the obstructed pilosebaceous unit in acne vulgaris (Shannon, 2020). Furthermore, we compared a comparison of the growth and metabolic state of *C. acnes* under two different conditions: glucose and glycerol. Glucose was considered as the control condition, while glycerol represented the major carbon source in the pilosebaceous unit (see Materials and Methods). This is due to the fact that human sebum primarily consists of triglycerides, fatty acids, squalene, and wax esters (Akaza et al., 2014), and *C. acnes* produces extracellular lipase enzymes that hydrolyzes the triglycerides present in sebum, leading to the release of glycerol as a nutrient source (Coenye et al., 2021). However, there is a lack of experimental evidence regarding the uptake of other sebum constituents by *C. acnes*. Therefore, glycerol is considered the primary endogenous carbon sources in human sebum, which aligns with the existing knowledge that glycerol serves as a major carbon source for the skin microbiome, facilitating growth and biosurfactant production (Saikia et al., 2012; Balasubramaniam et al., 2020). The simulation results showed that the growth rate under glycerol condition was 31.5% lower compared to glucose condition, and the production of acetate was negligible, while there was a 1.6-folds increase in propionate production rates observed in the glycerol condition (**Figure 3A**). These findings consistent with observations made in several *Propionibacterium* species (Liu et al., 2011; Wang and Yang, 2013; Zhang et al., 2015). The breakdown of carbon output from each source revealed that propionate exhibited the highest efflux in both conditions, while the contribution of CO_2_ to the total carbon output was lower in the glucose condition as expected (**Figure 3B**).

The resulting internal fluxes within the central metabolism and SCFA biosynthetic pathways clearly revealed that pyruvate kinase (PYK) in the last step of glycolysis and all reactions in the Wood-Werkman cycle have significantly higher fluxes in the glycerol condition compared to the glucose (up to 2.5-folds), while pyruvate dehydrogenase (PDH) is highly active in the glucose condition (up to 7.2-folds), resulting in higher acetate secretion coupled with CO_2_ production (**Figure 3C**). We further explored the Wood-Werkman cycle and PDH from a redox balance perspective by quantifying the turnover rates of energy cofactors such as ATP and NAD based on their flux-sum intensity values (see Materials and Methods). It should be noted that flux-sum can represent the metabolite pool size by summing up all incoming or outgoing fluxes associated with the metabolite (Chung and Lee, 2009). As a result, we observed a higher turnover rate of NAD (42.2%) in the glycerol-rich condition (**Figure 3D**), which is mainly contributed by glycerol-3-phosphate dehydrogenase (G3PD1) and malate dehydrogenase (MDH) reactions in glycolysis and TCA cycle, respectively. This observation is in good agreement with previous study on several *Propionibacterium* species, for example *P. jensenii*, (Liu et al., 2011; Wang and Yang, 2013; Zhang et al., 2015), which also possess the Wood-Werkman cycle to maintain cellular redox balance (Luna-Flores et al., 2018; Gonzalez-Garcia et al., 2020). Similarly, ATP turnover rate was increased by 20.4% in the glycerol due to the high ATP demand *via* glycerol kinase (GLYK) to consume the carbon source. Consequently, the cellular metabolism shifted towards ATP regeneration rather than cell growth, as indicated by the activation of PYK in glycolysis and the subsequent increase in the pyruvate pool which serves as a precursor for the metabolic reactions of the Wood-Werkman cycle. Overall, the simulation results suggest that in the context of acne vulgaris-associated skin conditions, *C. acnes* may utilize the Wood-Werkman cycle to replenish depleted NAD. This metabolic adaptation can lead to the overproduction of propionate, which in turn may trigger an inflammatory response in human skin.

### 3.3 Systematic identification of potential antimicrobial targets in *C. acnes* by gene essentiality analysis

**Figure 4:**
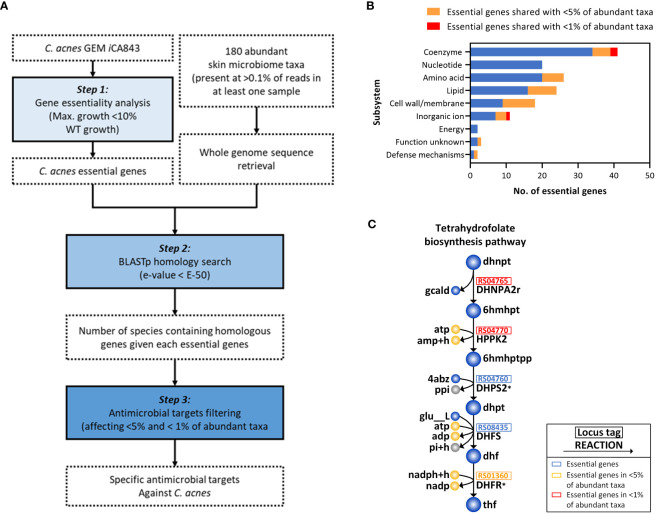
Systematic identification of potential anti-*C. acnes* targets. **(A)** Steps in systematic for identification of the specific antimicrobial candidate for *C. acnes.* **(B)** Classification of essential genes in various metabolic subsystems based on their functional roles. **(C)** Tetrahydrofolate biosynthetic pathway related to highly unique genes. The metabolic reactions inhibited by trimethoprim-sulfamethoxazole were indicated with an asterisk (*).

Considering that the current antibiotics used for acne vulgaris treatment that reduce *C. acnes* population may cause dysbiosis in the skin microbiome, which can lead to other skin diseases (Chien et al., 2019; Thompson et al., 2020), we presented a model-driven framework for systematically screening antimicrobial candidates and identifying promising targets which selectively suppress the growth of *C. acnes* with minimal effects on other skin microbiota (**Figure 4A**). Initially, using *i*CA843, we applied gene essentiality analysis to determine the genes which are crucial for the cell growth (see Materials and Methods), and found 117 essential genes (13.9%) out of 843 genes (step 1). With the list of abundant microbiome taxa (180 species) (Bewick et al., 2019) and their whole genome sequences collected from NCBI database, the number of species containing homologous genes given each essential gene for *C. acnes* were obtained *via* protein sequence similarity search using BLASTp (step 2). It is followed by narrowing down the gene list which are found only within less than 5% and 1% of abundant skin microbes in step 3, resulting in 23 ‘unique’ and 3 ‘highly unique’ candidates, respectively (**Supplementary Table 5**). In **Figure 4B**, we showed the distribution of essential genes including *C. acnes* specific antimicrobial targets (117 genes in total) in various metabolic subsystems which are classified based on their cluster of orthologous groups (COG) functional categories. The largest portion belongs to ‘coenzyme metabolism’ with two highly unique genes, indicating its high rigidity. Amino acids and nucleotide metabolisms have high number of essential genes, but most of them were not unique as expected since they are functionally conserved among bacterial species (Peregrín-Alvarez et al., 2009). Interestingly, 50% of the essential genes in the ‘lipid metabolism’ were found to be unique genes.

We further investigated biological mechanisms and pathways related to highly unique genes, which allowed us to identify promising *C. acnes* drug targets for the acne vulgaris treatment (**Figure 4C**). It should be noted that none of the highly unique candidates were homolog to human genome, and one of three genes have been discarded due to their functional ambiguity. The two highly unique genes encode dihydroneopterin aldolase (DHNPA2r) and 2-amino-4-hydroxy-6-hydroxymethyldihydropteridine diphosphokinase (HPPK2) which are involved in biosynthesis of tetrahydrofolate, a central cofactor in bacterial amino acid and nucleic acid metabolism (Tjong et al., 2022). In fact, a formate-tetrahydrofolate ligase enzyme, utilizing tetrahydrofolate as a substrate and producing 10-formyltetrahydrofolate, is present as a housekeeping gene in 72 strains of *C. acnes* (Kilian et al., 2012), indicating the essentiality of tetrahydrofolate for their survival. Thus, the current model-guided framework enabled us to identify two promising *C. acnes*-specific antimicrobial targets, the enzyme encoding DHNPA2r and HPPK2, which await further experimental validation.

## 4 Discussion

Despite the worldwide prevalence and severity of acne vulgaris, the pathogenic mechanisms of *C. acnes* under skin environment remain uncharacterized. Thus, in this study, we reconstructed a GEM of the virulent *C. acnes* strain, HL043PA1, to understand its pathogenic characteristics. The reconstructed GEM encompasses unique metabolic traits of *C. acnes*, including the propionate and acetate biosynthesis pathways as well as virulence-related metabolisms for coproporphyrin III and TAG lipase. Furthermore, the model successfully captures the innate production of SCFAs such as propionate and acetate without additional constraints on their effluxes. We also performed flux simulations to gain insight into its nutritional capabilities, specifically regarding carbon source utilization, and vitamin auxotroph. Then, we analyzed the metabolic states of *C. acnes* under endogenous carbon sources in human skin to elucidate its physiological behaviors. Interestingly, we observed that overproduction of propionate *via* the Wood-Werkman cycle is highly related to NAD regeneration under glycerol condition, indicating that inflammatory response induced by *C. acnes* may entail sebum-rich skin environment. Lastly, we utilized the model-driven framework to identify potential targets that selectively suppress the growth of *C. acnes* within skin microbiota.

Using *i*CA843, we elucidated physiological behaviors and metabolic states of *C. acnes*. However, several limitations exist in the current GEM. They include the limited availability of experimental data for carbon utilization and nutrient auxotroph as well as difficulties in *in vitro* culture due to its slow-growing properties, which can take up to two weeks under anaerobic condition (Elston et al., 2019). In addition, we were unable to observe the innate production of porphyrin without an additional constraint, although it is one of the main virulence factors produced at much higher levels in acne-associated strains compared to health-associated strains (Johnson et al., 2016). As porphyrin production has been reported to be regulated by the expression of the *deoR* repressor gene (Johnson et al., 2016; Barnard et al., 2020), this discrepancy may be attributed to gene regulation mechanisms, which require additional data such as transcriptomics to be integrated into the GEM. In future, *C. acnes* models can be further improved based on additional phenotypic and omics data such as transcriptome (Hastings et al., 2019; Jenior et al., 2020).

In our study, we identified potential antimicrobial targets for *C. acnes* using model-driven framework. In this framework, we utilized *i*CA843 to predict essential enzymes required for *C. acnes* growth, while minimizing off-target effects on the host microbiota *via* protein similarity search. Unlike previous model-guided studies on identifying potential drug targets for pathogens based on homolog of essential genes in their hosts (Plata et al., 2010; Sigurdsson et al., 2012; Koduru et al., 2020), our study focused on protein sequence similarity for both skin microbiome and human homologs. As a result, we identified two highly unique candidates, the enzymes encoding DHNPA2r and HPPK2, that have the potential to be used in the design and selection of effective antimicrobial inhibitors. In fact, the tetrahydrofolate biosynthetic pathway has been widely investigated for antimicrobial targets. For example, trimethoprim-sulfamethoxazole (Bactrim^®^) inhibits dihydropteroate synthetase (DHPS2) and dihydrofolate reductase (DHFR) within this pathway (Hooton, 2003). Furthermore, the *folB* gene, encoding DHNPA2r, has been identified as essential for *Mycobacterium tuberculosis* and recognized as a potential anti-tuberculosis drug target (Falcão et al., 2017). Therefore, the candidates proposed in this study hold promise as targets for the design and selection of effective *C. acnes*-specific antimicrobial inhibitors, which require further experimental validation.

In this study, we employed an e-value threshold of< 1 × 10^-50^ to determine the homology of essential proteins in *C. acnes* with other skin microbiota. However, it should be noticed that there is no gold standard for protein homology search, and the choice of threshold may vary depending on the analysis objectives and research scope. By using more lenient criteria, a smaller set of targets can be identified. For instance, when using an e-value threshold of<10^-30^, we observed that one of the two suggested targets, the enzyme encoding HPPK2, exhibited homology with proteins from four skin microbiota, while another target, DHNPA2r enzyme, showed homology with a protein from two skin microbiota. These observations emphasize the need to carefully specify the threshold criteria in line with the objectives and scope of identifying effective drug targets against *C. acnes*.

Arguably, *C. acnes* exhibits two faces as both pathogen and commensal, which is attributable to the metabolic diversity of RTs. Specifically, RT5 is strongly associated with acne, while RT6 is enriched in healthy skin, and RT1 is abundant in both acne and normal individuals (Fitz-Gibbon et al., 2013; Lomholt et al., 2017). In this regard, in order to investigate the metabolic variations among ribotypes, we additionally reconstructed GEMs of *C. acnes* ATCC6919 (RT1) and HL110PA3 (RT6) based on protein similarity search and *i*CA843 (see Material and Methods). Surprisingly, the ribotype-specific GEMs shared similar central metabolic pathways including glycolysis, pentose phosphate pathways and TCA cycle, with minor variations in cellobiose utilization and phenylalanine biosynthesis (see **Supplementary Table 6**). These metabolic similarities motivated us to further explore the potential genetic variations among ribotypes that may underlie the two faces of *C. acnes* strains. Thus, we conducted comparative genomic analysis (see Material and Methods), resulting in a total of 1,467 genes identified as core genes present in all the strains, and 58, 16, and 1 RT-specific genes in one of the RT1, 5, or 6, respectively. A heatmap of the dispensable genes in strains belonging to RT1, 5 and 6 are present in **Supplementary Image 1**. Among the RT5-specific genes, 6 were associated with virulence, including genes involved in toxin/antitoxin systems and endonucleases. Note that the rest were not functionally annotated. Therefore, functional regulations of the genes and unknown ribotype-specific genes should be incorporated into each RT-GEM, which will enable us to fully understand the distinct pathogenetic features among RTs.

Recent studies reported that microbial interactions shaping the composition of resident microbiota elicit notable influence in pathogenesis of skin disorders including acne vulgaris (Byrd et al., 2018; Ramasamy et al., 2019; Yang et al., 2022). For instance, *C. acnes* and *Staphylococcus* species (e.g., *S. epidermidis*), a predominant genus of skin microbiome, have antagonistic relationships by secreting SCFAs that inhibit the growth of each other (Nakamura et al., 2020; Ahle et al., 2022). In this regard, model-guided approach can be exploited to understand the roles of *C. acnes* and *S. epidermidis*, their relationship and skin community-level metabolic interactions during the development of acne vulgaris as similarly done for lactic acid bacteria strains (Shoaie et al., 2013; Koduru et al., 2022) and gut microbiome based on the models derived from AGORA (Magnúsdóttir et al., 2017), thereby providing a springboard for rational design of skin probiotics to restore healthy microbiome or to develop personalized treatment of acne vulgaris in future.

## 5 Conclusion

Our study provides insights into the metabolic characteristics behind several unique characteristics of *C. acnes*, including acne-associated SCFAs production and virulent pathways, through *in silico* analysis using *i*CA843. We simulated *C. acnes* behavior under glycerol, which resulted in overproduction of propionate related to pathogenesis of acne vulgaris. We also suggested the potential *C. acnes*-specific antimicrobial candidates that may minimize off-target effects to other skin microbes *via* the model-guided framework. Additional reconstruction of ribotype-specific GEMs and subsequent comparative genomics suggested that the *i*CA843 can also be applied to study metabolic differences between ribotypes and cross-feeding interactions with other skin microbes in near future.

## Data availability statement

The datasets presented in this study can be found in online repositories. The names of the repository/repositories and accession number(s) can be found in the article/**Supplementary Material**.

## Author contributions

S-KK, ML, YQL and S-YP wrote the original manuscript and revised the manuscript. ML and S-KK performed metabolic reconstruction and computational simulations. YQL, S-YP and D-YL guided and helped with the in-silico modeling. S-YP and S-KK analyzed and interpreted overall data. S-KK, ML, and YQL visualized the data. HJL and MR analyzed and interpreted genome data. YK, JYS, SHY, SJH, NGK and CHL reviewed the manuscript. S-YP and D-YL supervised the study. D-YL conceptualized the study and revised the manuscript. All authors read and approved the final version of manuscript.

## Supplementary material

Supplementary Data Sheet 2 The genome-scale metabolic model of Cutibacterium acnes (iCA843).

Supplementary Data Sheet 3 The MEMOTE score of iCA843.
