# Supplementary material for: Genome-scale metabolic modeling and in silico analysis of opportunistic skin pathogen Cutibacterium acnes
Source: Front Cell Infect Microbiol. 2023 Jul 13;13:1099314. doi: 10.3389/fcimb.2023.1099314 (PMC10374032; doi:10.3389/fcimb.2023.1099314)
Supplement: Supplementary file 1 [file DataSheet_1.docx]

Supplementary Data Sheet 1. Biomass equation formulation

**Macromolecular composition**

The cell wall contents to dry cell weight of *P. rubrum* and *P.* *peterssonii* were estimated to be 16 to 20% (Allsop, 1963). Among cell wall contents, the composition of lipomannan was adopted from data of *Propionibacterium freudenreichii* (Sutcliffe & Shaw, 1993), using, with a value of 0.72% of total dry cell weight. In addition, composition of total carbohydrate among cell wall contents is estimated by average of relative weight percentage of carbohydrate from two studies of *Propionibacterium species* (Kamisango et al., 1982; Sutcliffe & Shaw, 1993), which is 32.5% (McCubbin et al., 2020). The lipid contents of *P. shermanii* and *P. acidipropionici* were estimated to be 3% in two separate studies. However, *Propionibacterium*-specific total lipid content and cell wall composition to dry weight exists in literature, protein, DNA, RNA small molecule and ash compositions of *C. acnes* were not quantified. Therefore, biomass composition data of gram-positive actinobacteria *Streptomyces coelicolor* (Borodina et al., 2005) was used for biomass composition.

Table 1. Cellular composition

| **Amino acids** | **Composition of S. coelicolor (g/gDCW)** | **Propionibacterium specific composition (g/gDCW)** | **Corrected composition (g/gDCW)** | **Average protein composition  (mmol/g)** |
| --- | --- | --- | --- | --- |
| Protein | 0.412 |  | 0.43162 | 0.43162 |
| DNA | 0.036 |  | 0.03771 | 0.03771 |
| RNA | 0.167 |  | 0.17495 | 0.17495 |
| Phospholipid | 0.045 | 0.03 | 0.03 | 0.03 |
| Cell wall | 0.22 | 0.2 | 0.2 | 0.2 |
| Ash | 0.09 |  | 0.09429 | 0.09429 |
| Small molecules | 0.03 |  | 0.03143 | 0.03143 |

Biomass reaction becomes (in mmol for synthesis of 1 g of biomass)

**0.432 PROTEIN + 0.0377 DNA + 0.175 RNA + 0.03 PHOSPHOLIPID + 0.135 PEPTIDOGLYCAN + 0.0578 CARBOHYDRATE + 0.0072 LIPOMANNAN + 0.0314 SMALL MOLECULES -> BIOMASS**

**Protein Composition**

Data on protein composition of C. acnes were estimated from the protein sequence of C. acnes. The molecular weight of the amino acids given in the following table excludes the weight of the water molecule to account for water excretion during peptide bond formation.

Table 2. Protein Composition

| **Amino acids** | **Molar ratio (mol/mol)** | **MW (g/mol)** | **Protein composition (mmol/g)** |
| --- | --- | --- | --- |
| Alanine | 0.11 | 71.09 | 1.02552 |
| Arginine | 0.07 | 156.20 | 0.62825 |
| Asparagine | 0.03 | 114.12 | 0.23097 |
| Aspartate | 0.06 | 115.10 | 0.58205 |
| Cysteine | 0.01 | 103.16 | 0.10163 |
| Glutamate | 0.03 | 128.15 | 0.28641 |
| Glutamine | 0.05 | 129.13 | 0.48966 |
| Glycine | 0.09 | 57.07 | 0.78531 |
| Histidine | 0.03 | 137.16 | 0.23097 |
| Isoleucine | 0.05 | 113.18 | 0.46195 |
| Leucine | 0.09 | 113.18 | 0.86846 |
| Lysine | 0.03 | 128.19 | 0.26793 |
| Methionine | 0.03 | 131.21 | 0.23097 |
| Phenylalanine | 0.03 | 147.19 | 0.26793 |
| Proline | 0.05 | 97.13 | 0.48042 |
| Serine | 0.06 | 87.09 | 0.56357 |
| Threonine | 0.06 | 101.12 | 0.58205 |
| Tryptophan | 0.02 | 186.23 | 0.14782 |
| Tyrosine | 0.02 | 163.19 | 0.18478 |
| Valine | 0.09 | 99.15 | 0.82226 |

The energy requirement for polymerization of protein is 39.78 mmol ATP/g protein.

Protein biosynthesis equation becomes (in mmol for synthesis of 1 g of protein)

**1.026 ala__L + 0.628 arg__L + 0.231 asn__L + 0.582 asp__L + 0.102 cys__L + 0.286 gln__L + 0.49 glu__L + 0.785 gly + 0.231 his__L + 0.462 ile__L + 0.868 leu__L + 0.268 lys__L + 0.231 met__L + 0.268 phe__L + 0.48 pro__L + 0.564 ser__L + 0.582 thr__L + 0.148 trp__L + 0.185 tyr__L + 0.822 val__L + 39.78 atp -> 39.78 adp + 39.78 pi _ PROTEIN_c**

**DNA Composition**

The composition of DNA of *C. acnes* was acquired from genome sequence of *C. acnes* HL043PA1 available in NCBI. The molecular weight of nucleotide monophosphate excludes weight of a water molecule as it is lost during esterification.

Table 3. DNA composition

| **Components** | **Molar ratio (mol/mol)** | **MW (g/mol)** | **DNA (mmol/g)** |  |
| --- | --- | --- | --- | --- |
|  |  |  |  |  |
| dAMP | 0.199 | 313.2 | 0.644057 |  |
| dTMP | 0.199 | 304.2 | 0.647294 |  |
| dCMP | 0.301 | 289.2 | 0.974177 |  |
| dGMP | 0.301 | 329.2 | 0.970941 |  |

The energy requirement of DNA polymerization is 4.44 ATP/g DNA

DNA biosynthesis equation becomes (in mmol for synthesis of 1 g of DNA)

**0.644 dATP + 0.647 dTTP + 0.974 dCTP c + 0.971 dGTP + 4.44 ATP -> 3.236 ppi + 4.44 ADP + 4.44 pi + DNA**

**RNA composition**

We assumed that the RNA composition consisted of 5% mRNA, 75% rRNA and 20% tRNA (molar). The mRNA composition was estimated from DNA sequence. The rRNA sequence was calculated from 16S, 23S and 5S ribosomal RNA sequences. The tRNA sequence was calculated from sequences of amino acid transporting RNAs. All sequences were collected from NCBI database.

Table 4. RNA composition

| **Components** | **mol/mol RNA** | | | **MW**  **(g/mol)** | **Molar ratio**  **(mol/mol)** | **RNA**  **(mmol/g)** |
| --- | --- | --- | --- | --- | --- | --- |
|  | **mRNA** | **rRNA** | **tRNA** |  |  |  |
|  | **5%** | **75%** | **20%** |  |  |  |
| AMP | 0.199 | 0.223 | 0.199 | 345.2 | 0.217 | 0.68 |
| CMP | 0.301 | 0.352 | 0.335 | 305.2 | 0.346 | 1.085 |
| GMP | 0.300 | 0.211 | 0.295 | 306.2 | 0.233 | 0.729 |
| UMP | 0.200 | 0.213 | 0.171 | 329.2 | 0.204 | 0.64 |

The energy requirement of RNA polymerization is 1.25 ATP/g RNA

RNA biosynthesis equation becomes (in mmol for synthesis of 1 g of RNA)

**0.680 ATP + 1.085 CTP + 0.729 GTP + 0.640 UTP + 1.25 ATP -> 1.25 ADP + 1.25 pi + RNA + 3.135 ppi**

**Phospholipids composition.**

The composition of phospholipids was adopted from g data of *Propionibacterium freudenreichii* (Sutcliffe & Shaw, 1993).

Table 5. Phospholipid composition

| **Components** | **Relative amounts** | **Proportions (mol/mol)** | **Phospholipids (g/mol)** | **Phospholipids (mmol/g)** |
| --- | --- | --- | --- | --- |
| Cardiolipin | 3 | 0.2727272727 | 358.8379 | 0.299 |
| Phosphatidylglycerol | 2 | 0.1818181818 | 127.8016 | 0.199 |
| Phosphatidylinositol | 2 | 0.1818181818 | 143.8073 | 0.199 |
| Lyso-phosphatidylinositol | 1 | 0.09090909091 | 51.19182 | 0.100 |
| Phosphatidylethanolamine | 1 | 0.09090909091 | 61.17327 | 0.100 |
| Acylphosphatidylglycerol | 2 | 0.1818181818 | 169.4218 | 0.199 |

Phospholipid biosynthesis equation becomes (in mmol for synthesis of 1 g of phospholipid)

**0.299 Cardiolipin + 0.199 Phosphatidylglycerol + 0.199 Phosphatidylinositol + 0.100 Lyso-phosphatidylinositol + 0.100 Phosphatidylethanolamine + 0.199 Acylphosphatidylglycerol → PHOSPHOLIPID**

Table 6. Molecular weights of phospholipid components

| **Constituent** | **Backbone (g/mol)** | **# of fatty acids residues** | **Total (g/mol)** |
| --- | --- | --- | --- |
| Cardiolipin | 332.179 | 4 | 1315.739 |
| Phosphatidylglycerol | 211.1286 | 2 | 702.9086 |
| Phosphatidylinositol | 299.16 | 2 | 790.94 |
| Lyso-phosphatidylinositol | 317.22 | 1 | 563.11 |
| Phosphatidylethanolamine | 181.126 | 2 | 672.906 |
| Acylphosphatidylglycerol | 194.15 | 3 | 931.82 |

The average of fatty acid composition of 27 Corynebacterium acnes isolates was adopted from fatty acid compositions of *Corynebacterium acnes* (Moss et al., 1967).

Table 7. Fatty acids composition

| **Fatty acids** | **Ratio (g/g)** | **MW (w/o H) (g/mol)** | **Total fatty acids (mmol/g)** |
| --- | --- | --- | --- |
| C11 | 0.02256447 | 185.29 | 0.121779 |
| C12 | 0.048710602 | 199.3178 | 0.244387 |
| C13 | 0.01969914 | 213.348 | 0.092333 |
| C14 | 0.078080229 | 227.37 | 0.343406 |
| C15Br | 0.359240688 | 241.3975 | 1.488171 |
| C15 | 0.107449857 | 241.3975 | 0.445116 |
| C16:1 | 0.022922636 | 253.414 | 0.090455 |
| C16 | 0.126074499 | 271.42 | 0.4645 |
| C17Br | 0.092406877 | 269.45 | 0.342946 |
| C17 | 0.018624642 | 269.45 | 0.069121 |
| C18:1 | 0.059097421 | 281.47 | 0.20996 |
| C18 | 0.030802292 | 283.48 | 0.108658 |
| C20 | 0.014326648 | 311.5304 | 0.045988 |

Molecular weight of fatty acids presented above is molecular weight without proton. The average molecular weight is 245.89 g/mol.

**Peptidoglycan composition**

The composition of peptidoglycan was taken from data of *P. acnes* strain C7 (Kamisango et al., 1982). The blocks of glycan chain are provided with activated form, UDP-N-acetylmuramic acid and UDP-N-acetylglucosamine, thereby only energy for pentapeptide formation is needed (ATP per mol of amino acid connected). The ratio of L-alanine to total alanine was approximately 1 :1.3. D-alanine combines to the growing amino acids chain as D-alanyl-D-alanine.

Table 8. Peptidoglycan composition

| **Components** | **Peptidoglycan (mol/mol)** | **MW (g/mol)** | **Peptidoglycan (mmol/g)** |
| --- | --- | --- | --- |
| Glutamic acid | 0.17921 | 129.13 | 1.245 |
| Glycine | 0.13978 | 39.07 | 0.971 |
| Alanine | 0.21326 | 71.09 | 1.481 |
| Diaminopimelic acid | 0.15412 | 154.2 | 1.071 |
| N-acetylmuramic acid | 0.17563 | 275.27 | 1.22 |
| N-acetylglucosamine | 0.13799 | 203.21 | 0.959 |

The energy required for polymerization is 4.769 mmol ATP/g peptidoglycan. The peptidoglycan biosynthesis equation becomes (in mmol for synthesis of 1 g of peptidoglycan)

**1.22 N-acetylmuramic acid + 0.959 UDP-N-acetylglucosamine + 0.877 L-Alanine + 0.342 Ala-Ala + 1.071 LL-2,6-Diaminopimelate + 1.245 D-Glutamate + 0.971 Glycine + 4.769 ATP → 4.769 ADP + 4.769 pi + 0.342 D-alanine + Peptidoglycan**

**Polysaccharide composition**

The composition of polysaccharide was adopted from data of *P. acnes* strain C7 (Nagaoka et al., 1985). The blocks of polysaccharide are provided with activated form, thereby no additional ATP consumption is needed during polymerization. Molecular weight was subtracted from the weight of water to take the bond formation into account.

Table 9. Polysaccharide composition

| **Component** | **Molar ratio** | **MW (g/mol)** | **Polysaccharide  (mmol/g)** |
| --- | --- | --- | --- |
| Glucose | 0.1875 | 162.156 | 0.912 |
| Galactose | 0.1875 | 162.156 | 0.912 |
| Mannose | 0.0625 | 162.156 | 0.304 |
| N-acetyl-galactosamine | 0.1875 | 203.21 | 0.912 |
| 2,3-diacetamido-2,3-dideoxymannuronate | 0.375 | 257.24 | 1.825 |

Polysaccharide biosynthesis equation becomes (in mmol for synthesis of 1 g of polysaccharide)

**0.912 UDP-Glucose + 0.912 UDP-Galactose + 0.304 UDP-Mannose + 0.912 UDP-N-acetyl-galactosamine + 1.825 UDP-2,3-diacetamido-2,3-dideoxymannuronate → POLYSACCHARIDE + 4.561 UDP + 0.304 GDP**

**Lipomannan composition**

In *C. acnes*, lipoglycan is present rather than LTA (Whale et al., 2004). The lipomannan composition was adopted from data of *Propionibacteria* (Sutcliffe & Shaw, 1993). A mannan of approximately 30 mannose residues was proposed to be anchored to cell membrane. The molecular weight of mannose given in the following table excludes the weight of the water molecule to account for water excretion during bond formation. The blocks of glycan chain are provided with activated form, GDP-mannose, thereby no extra energy is needed.

Table 10. Lipomannan composition

| **Component** | **Molar ratio** | **MW (g/mol)** | **Lipomannan (mmol/g)** |
| --- | --- | --- | --- |
| Mannose | 30 | 162.156 | 6.173 |

Lipomannan biosynthesis equation becomes (in mmol for synthesis of 1 g of lipomannan)

**6.173 GDP-mannose → 6.173 GDP + 6.173 H + LIPOMANNAN**

**Small molecules composition**

The composition of small molecules was adopted from data of *E. coli* (Orth et al., 2011). Electron transport chain components such as protoheme, siroheme, 2Fe-2S and 4Fe-4S iron sulfur clusters, and dehydrogenated menaquinol-9 and essential vitamins and inorganic ions were included in small molecules pools.

Table 11. Small molecules composition

| **Component** | **Composition  (g/g)** | **MW (g/mol)** | **Small molecules (mmol/g)** |
| --- | --- | --- | --- |
| TPP | 0.030564899 | 423 | 0.07226 |
| FAD | 0.030564899 | 783 | 0.03904 |
| Riboflavin | 0.030564899 | 376 | 0.08129 |
| NAD | 0.030564899 | 662 | 0.04617 |
| NADH | 0.030564899 | 663 | 0.0461 |
| NADP | 0.030564899 | 741 | 0.04125 |
| NADPH | 0.030564899 | 742 | 0.04119 |
| CoA | 0.030564899 | 764 | 0.04001 |
| Pyridoxal phosphate | 0.030564899 | 246 | 0.12425 |
| BIOT | 0.030564899 | 243 | 0.12578 |
| Niacin(B3) | 0.030564899 | 123.109 | 0.24827 |
| pantothenate(B5) | 0.030564899 | 219.23 | 0.13942 |
| 10-Formyltetrahydrofolate | 0.030564899 | 471 | 0.06489 |
| 5-Methyltetrahydrofolate | 0.030564899 | 457 | 0.06688 |
| 5-10-Methylenetetrahydrofolate | 0.030564899 | 455 | 0.06718 |
| Tetrahydrofolate | 0.030564899 | 443 | 0.069 |
| Calomide | 0.030564899 | 1579 | 0.01936 |
| Menaquinol-9(4H) | 0.030564899 | 788 | 0.03879 |
| S-Adenosyl-L-methionine | 0.030564899 | 399 | 0.0766 |
| Siroheme | 0.030564899 | 908 | 0.03366 |
| Heme | 0.030564899 | 614 | 0.04978 |
| 2Fe2S | 0.030564899 | 532 | 0.05745 |
| 4Fe4S | 0.030564899 | 1064 | 0.02873 |
| NH3 | 0.01238 | 18 | 0.39427 |
| Cl- | 0.00495 | 92 | 0.15764 |
| Co2+ | 0.00002 | 59 | 0.00064 |
| Fe2+ | 0.00639 | 56 | 0.2035 |
| fe3 | 0.00743 | 56 | 0.23662 |
| Mn2+ | 0.00066 | 55 | 0.02102 |
| Mg | 0.00825 | 24 | 0.26274 |
| K+ | 0.18569 | 39 | 5.91369 |
| Sulfate | 0.00413 | 96 | 0.13153 |

Small molecules biosynthesis equation becomes (in mmol for synthesis of 1 g of small molecules)

**0.072 TPP + 0.039 FAD + 0.081 Riboflavin + 0.046 NAD + 0.046 NADH + 0.041 NADP + 0.041 NADPH + 0.04 CoA + 0.124 Pyridoxal phosphate + 0.126 BIOT + 0.248 nac + 0.139 pnto__R + 0.065 10-Formyltetrahydrofolate + 0.067 5-Methyltetrahydrofolate + 0.067 5-10-Methylenetetrahydrofolate + 0.069 Tetrahydrofolate + 0.019 Calomide + 0.039 Menaquinol-9(4H) + 0.077 S-Adenosyl-L-methionine + 0.034 Siroheme + 0.05 Heme + 0.057 2Fe2S + 0.029 4Fe4S + 0.394 NH3 + 0.158 Cl- + 0.001 Co2+ + 0.204 Fe2+ + 0.237 fe3 + 0.021 Mn2+ + 0.263 Mg + 5.914 K+ + 0.132 Sulfate -> SMALL MOLECULE**

**References**

ALLSOP J WORK, E. (1963). Cell walls of Propionibacterium species: fractionation and composition. *The Biochemical Journal*, *87*(3), 512–519. https://doi.org/10.1042/BJ0870512

Borodina, I., Krabben, P., & Nielsen, J. (2005). Genome-scale analysis of Streptomyces coelicolor A3(2) metabolism. *Genome Research*, *15*(6), 820–829. https://doi.org/10.1101/GR.3364705

KAMISANGO, K., SAIKI, I., TANIO, Y., OKUMURA, H., ARAKI, Y., SEKIKAWA, I., AZUMA, I., & YAMAMURA, Y. (1982). Structures and Biological Activities of Peptidoglycans of Listeria monocytogenes and Propionibacterium acnes12. *The Journal of Biochemistry*, *92*(1), 23–33. https://doi.org/10.1093/oxfordjournals.jbchem.a133918

McCubbin, T., Gonzalez-Garcia, R. A., Palfreyman, R. W., Stowers, C., Nielsen, L. K., & Marcellin, E. (2020). A Pan-Genome Guided Metabolic Network Reconstruction of Five Propionibacterium Species Reveals Extensive Metabolic Diversity. *Genes*, *11*(10), 1–26. https://doi.org/10.3390/GENES11101115

Moss, C. W., Dowell, V. R., Lewis, V. J., & Schekter, M. A. (1967). Cultural Characteristics and Fatty Acid Composition of Corynebacterium acnes. *Journal of Bacteriology*, *94*(5), 1300. https://doi.org/10.1128/JB.94.5.1300-1305.1967

NAGAOKA, M., KAMISANGO, K., FUJII, H., UCHIKAWA, K., SEKIKAWA, I., & AZUMA, I. (1985). Structure of Acidic Polysaccharide from Cell Wall of Propionibacterium acnes Strain C71. *The Journal of Biochemistry*, *97*(6), 1669–1678. https://doi.org/10.1093/oxfordjournals.jbchem.a135225

Orth, J. D., Conrad, T. M., Na, J., Lerman, J. A., Nam, H., Feist, A. M., & Palsson, B. (2011). A comprehensive genome-scale reconstruction of Escherichia coli metabolism--2011. *Molecular Systems Biology*, *7*. https://doi.org/10.1038/MSB.2011.65

Sutcliffe, I. C., & Shaw, N. (1993). The Phospholipids of Propionibacterium freudenreichii: Absence of Phosphatidylinositol Mannosides. *Systematic and Applied Microbiology*, *16*(1), 9–12. https://doi.org/10.1016/S0723-2020(11)80243-2

Whale, G. A., Sutcliffe, I. C., Morrisson, A. R., Pretswell, E. L., & Emmison, N. (2004). Purification and characterisation of lipoglycan macroamphiphiles from Propionibacterium acnes. *Antonie van Leeuwenhoek 2004 86:1*, *86*(1), 77–85. https://doi.org/10.1023/B:ANTO.0000024911.67625.27
