# Supplementary figures and images for: Genome-scale metabolic modeling and in silico analysis of opportunistic skin pathogen Cutibacterium acnes
Source: Front Cell Infect Microbiol. 2023 Jul 13;13:1099314. doi: 10.3389/fcimb.2023.1099314 (PMC10374032; doi:10.3389/fcimb.2023.1099314)

# Supplementary Image

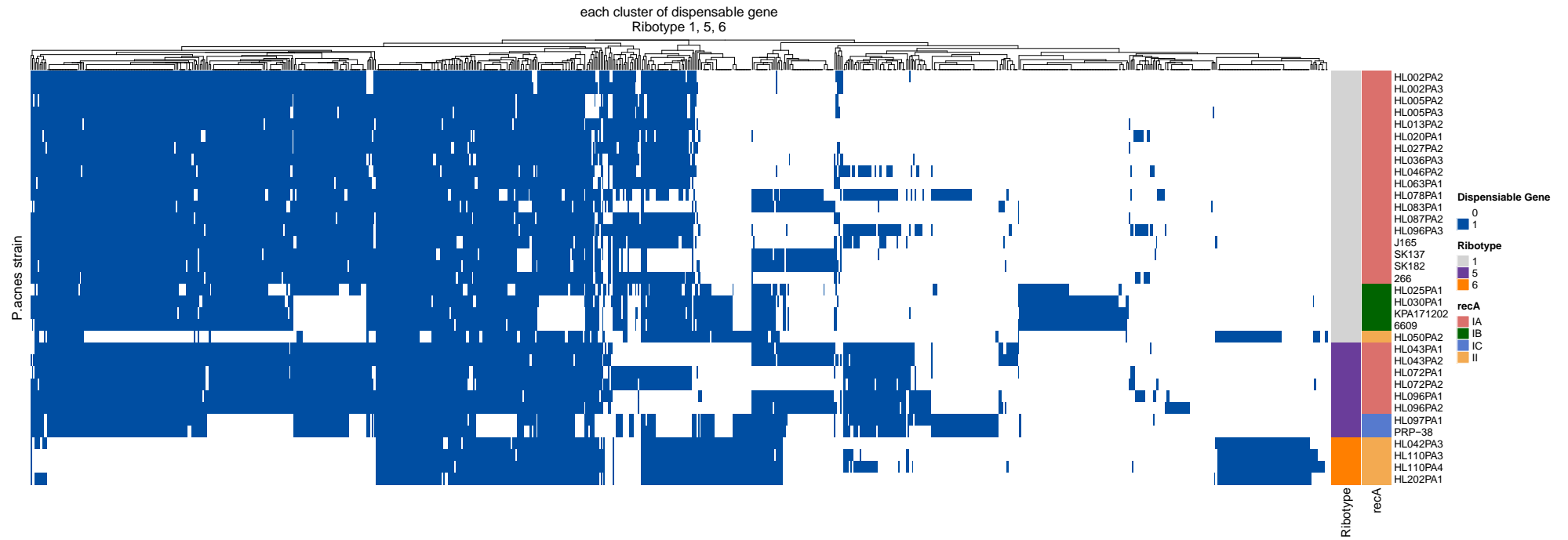

**Image S1** Heatmap of the dispensable genes in the strains of ribotype 1, 5, and 6.

Supplement: Supplementary file 4 [file Image_1.pdf]
